# Japanese Resident Physicians' Attitudes, knowledge, and Perceived Barriers on the Practice of Evidence Based Medicine: a Survey

**DOI:** 10.1186/1756-0500-4-374

**Published:** 2011-09-28

**Authors:** Risahmawati RM Risahmawati, Sei SE Emura, Tomoko TN Nishi, Shunzo SK Koizumi

**Affiliations:** 1 Department of General Medicine, Saga University Graduate School of medicine, Saga University, Saga 849-8501, Japan; 2 Center for Graduate Medical Education Development and Research, Faculty of Medicine, Saga University, Saga 849-8501, Japan; 3 Department of General Medicine, Faculty of Medicine, Saga University, Saga 849-8501, Japan; 4 Faculty of Medicine and Health Sciences, Syarif Hidayatullah State Islamic University, Jl. Ir. H. Juanda No.95 Ciputat 15412 Jakarta, Indonesia

## Abstract

**Background:**

Evidence based medicine plays a crucial role as a tool that helps integrate research evidence into clinical practice. However, few reports have yet to examine its application in daily practice among resident physicians in Japan. The aim of this study was to assess the attitudes towards and knowledge of EBM among resident physicians in Japanese and determine perceived barriers to its use.

**Findings:**

A cross-sectional, self-administered anonymous questionnaire was distributed to 60 resident staffs at Saga University Hospital in Japan.

Forty residents completed and returned the questionnaire. Fifty four percent of respondents understood the basic terminology of EBM, 3% could explain this to others, and 41% indicated they would like to understand the terminology more. Thirteen percent admitted having a good understanding of EBM basic skills. Fifty respondents indicated having read EBM sources, but only 3% indicated that they use these sources in clinical decision making. The most prominent barriers of EBM application revealed in this study were insufficient time to access the sources, a lack of native language references, and insufficient basic EBM skills, but not scepticism about the EBM concept.

**Conclusions:**

In general, respondents positively welcomed EBM, and moderately understood and knew basic EBM skill; however, barriers in its application were shown to exist.

## Background

It is generally accepted that the more experience a physician has the better the quality of health care delivery. However, recent studies have shown that there is in fact an inverse relationship between the numbers of years a physician has been in practice and the quality of care provided [1]. This is quite surprising, being a stark contrast to the entire general assumptions of physicians and health services users. Moreover, this is also a potentially dangerous finding for patients if appropriate quality of health care is being affected, and for this reason quality improvement intervention is necessary [1]. There are various reasons for the above finding. For example, a physician's "toolkit" is created during training and may be not updated regularly. In addition, practice innovations that involve theoretical shifts may be harder to incorporate into the practice of physicians who trained a long time ago.

Evidence-based medicine (EBM) defined as the judicious use of the best current evidence in making decisions about the care of the individual patient. EBM is mean to integrate clinical expertise with the best available research evidence and patient values. EBM was initially proposed by Dr. David Sackett and colleagues at McMasters University in Ontario, Canada [2,3].

Thus, EBM has arisen as a new paradigm for medical practice; however, certain barriers to its use and practice remain [4-6]. For example, in practice, it is not always easy to apply the principles of EBM. Several factors attribute to these barriers such as misinterpretation, difficulty in teaching EBM and this results in the lack of training module in EBM, lack of time to access evidence based medicine source and the attitude of the physician themselves [4-8].

## Objectives

The objective of this study was to assess the attitudes toward and knowledge about EBM among residents and their perceived barriers to its use.

## Methods

We assessed residents attitudes towards EBM in a cross-sectional study conducted in Saga University Teaching Hospital, Japan. The participants of the survey are 60 participants, all the resident in first and second year residency. Among the entire participant, 40 participants returned the filled questionnaire. Questionnaire were given in one package of letter contain main questionnaire, inform consent, and respondents data. Incentive for respondent was not available. Reminder was sent by email, in some cases verbal reminder also given.

### Questionnaire Design and Survey Sample

The tool of data collection was a self-administered questionnaire consisted of closed question about the respondent's attitudes toward daily clinical decision making, respond to EBM, knowledge of and preference to different information sources, and possible barriers to EBM application was used for data collection (Table 1). We provided blank spaces for respondents to give their opinion freely.

**Table 1:** Structure of the questionnaire

Aspect of assessment	Questionnaire Content
Attitude	Question 1. Preference to information sources during clinical decision making
	Question 2. Opinion and attitude towards EBM
Knowledge	Question 3. Familiarity and use of electronic EBM sources
	Question 4. Knowledge of methodological terminology
	Question 5. Self-rated confidence in EBM skills
Self-perceived barrier	Question 6. Self-perceived barriers to EBM application

The questionnaire (Additional File 1) was derived from a previous study conducted in Saga University teaching hospital and a questionnaire adapted from previous studies in other countries [4,5,9,10]. To measure residents' attitudes towards EBM, knowledge and self-perceived barrier to implement EBM we used the Likert Scale.

This research was approved by the institutional review board of Saga University Hospital.

### Data Analysis

The statistical package for social science (SPSS) version "16" was used for data entry and analysis. Statistical tests were performed to determine the correlation between variables and to compare one group with another.

## Result

Responses were received from 40 of 60 residents giving a response rate of 67%. The demographic characteristic of the respondents shown in table 2.

**Table 2:** Characteristic of respondents

Characteristic	Description	Number (%) (n = 40)
Sex	Male	21 (52.5)
	Female	19 (47.5)
Age (years)	24	10 (25)
	25	13 (32.5)
	26	14 (35)
	27	2 (5)
	35	1(2.5)
Department	Surgery	9 (22.5)
	Non-Surgery	31 (77.5)
Working hours	< 40	4 (10)
	40-80	30 (75)
	> 81 hours	4 (10)
Internet access at work	Yes	30 (75)
	No	10 (25)

### Attitudes towards EBM

The attitudes towards EBM were assessed by two points, preferences to information sources during clinical decision making and attitudes toward EBM (Question 1 and 2).

**Figure 1:**
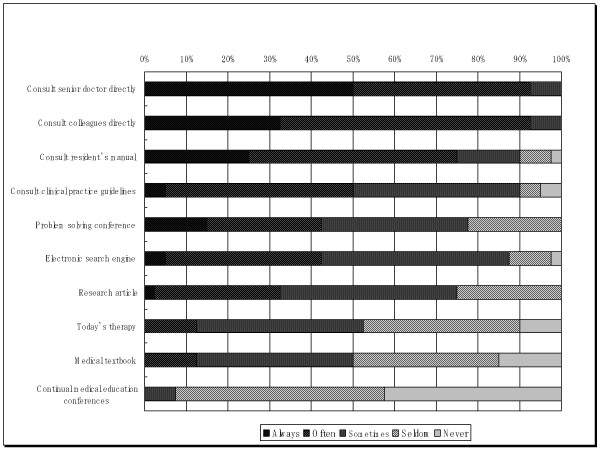
**Preferences to different information sources during clinical decision making**.

Figure 1 presents the preferences to each information source accessed by respondents. Presentation of sources was based on the rank of utility. Personal communication between colleagues and/or senior doctors was shown to be the most popular method of daily clinical practice problem solving while presenting problems at regular medical education conferences was rarely used to help obtain answers to clinical problems. Personal consultation seems to be the easiest and most comfortable way for residents to find answer during clinical practice, although on the other hand, expert opinion can sometimes be inferior to new scientific evidence. Both scientific evidence and medical opinions, however, have their own limitations. For example, as previously noted, many medical practices lack scientific evidence and, when available, often lack internal and external validity [11]. Overall, these findings show that respondents use both EBM and non-EBM methods to find answers during daily clinical problem solving.

**Figure 2:**
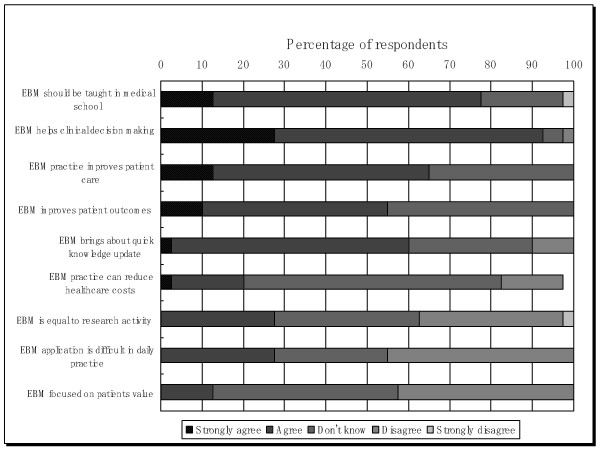
**Respondents' respond and attitudes toward evidence based medicine**.

We asked respondent about their opinion toward EBM practices. Positive respond toward EBM were seen in the statement EBM practices improves patient care (65%); EBM helps clinical decision making (92.5%); EBM brings about quick knowledge update (60%); EBM should be taught in medical school (77.5%); and only 27.5% respondent agree that EBM difficult to be applied in daily practices. Although many respondent answer don't know that EBM practice improves patients outcome (45%), EBM practices can reduce healthcare cost (62.5%), focus on patient value (45%), In one side respondents' respond to EBM practice positively while in another side negative respond to EBM practice were also noticeable. overall positive respond to EBM are the predominant answer. Figure 2 presents respondents' respond and attitudes toward evidence based medicine.

### Knowledge about EBM

To assess the knowledge to EBM we asked three points, familiarity to electronic EBM sources, understanding of methodological terminology and self-rated confidence in EBM Skill.

**Figure 3:**
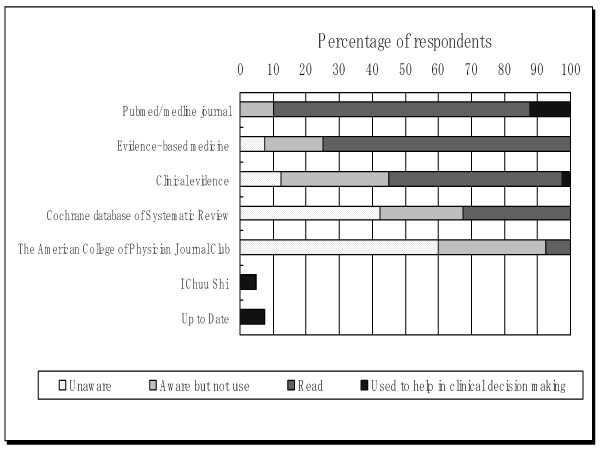
**Familiarity and use of electronic EBM sources**.

We asked several questions regarding respondents' knowledge of EBM and use of EBM sources. Questions concerned respondents' familiarity and usage of electronic EBM resources, knowledge of methodological terminology, and self-judgment of their confidence in applying EBM skills. Scoring values were given to each question as follows: score 1, unaware; 2, aware but do not use; 3, read; and 4, have used in clinical decision making. The average of these scores was then used in the statistical tests. Figure 3 presents familiarity and use of electronic EBM sources. Presentation of electronic EBM sources was based on the rank of utility.

Among all of the EBM resources indicated in this survey, only the Pubmed/Medline Journal and Clinical evidence from the BJ publishing group were indicated as having been used during clinical decision making. Other electronic EBM sources referred to were "up to date" and "I Chuu Shi" (a Japanese search engine).

Statistic tests relevant to each data-set were performed to compare and determine the relationship among variables. From these tests, we obtained the following results: there was no significant correlation between years of residency and familiarity and use of electronic EBM resources (P value for Spearman's correlation test = 0.196); residents in their first and second years did not differ in their use of EBM sources. Familiarity and use of electronic EBM resources did not differ between male and female residents (P value for Mann Whitney test = 0.226). Residents with internet access at work compared to those without internet access, had no effect on familiarity and use of electronic EBM resources (P value for Unpaired T test = 0.138).

### Knowledge of Methodological Terminology

We also asked respondents about their knowledge of methodological terminology frequently used in EBM papers. None of the respondents confessed to understanding and having the ability to explain to others about different terminologies examined.

Respondents' personal answers were classified into four categories, and gradual scoring values were given to each as follows: 1 for "it would not be helpful for me to understand"; 2, "don't understand but would like to"; 3, "some understanding"; and 4, "understand and could explain to others". Table 3 presents respondents' understanding of methodological terminology, and Table 4 presents Terms that respondents indicated wanting to know more about.

**Table 3:** Residents' Knowledge of Methodological Terminology [10].

**No**.	Terminology	Mean score
**1.**	Odds ratio	3.33
**2.**	Confidence interval	2.95
**3.**	Publication bias	2.75
**4.**	Sample bias	2.65
**5.**	Sensitivity and specificity	2.63
**6.**	Absolute risk	2.55
**7.**	Meta analysis	2.53
**8.**	Relative risk	2.5
**9.**	Clinical effectiveness	2.33
**10.**	Systematic review	2.3
**11.**	Number need to treat	1.85

**Table 4:** Terms that respondents indicated wanting to know more about

No.	Terminology	N (%)
**1.**	Number need to treat	32(80)
**2.**	Systematic review	29(72.5)
**3.**	Clinical effectiveness	27(67.5)
**4.**	Absolute risk	20(50)
**5.**	Meta analysis	19(47.5)
**6.**	Relative risk	18(45)
**7.**	Publication bias	12(30)
**8.**	Odds ratio	8(20)
**9.**	Sample bias	8(20)
**10.**	Confidence interval	5(12.5)
**11.**	Sensitivity and specificity	2(5)

In this study, respondents reported insufficient knowledge of methodological EBM terms but at the same time, the majority of respondents' showed enthusiasm to learn more (table 3 and table 4).

There was no significant correlation between the year of residency and knowledge of methodological terminology (p value of Spearman's correlation test = 0.467), and no significant correlation between familiarity and use of electronic EBM sources and knowledge of methodological terminology (P value for Pearson's correlation test was 0.729). Male and female did not differ in their knowledge of methodological terminology (P value for Mann Whitney's comparison test = 0.686). In addition, there was no significant mean difference in knowledge of methodological terminology between residents with internet access at work and those without (P value for unpaired T test = 0.686).

### Self-Rated Confidence in EBM Skill

**Figure 4:**
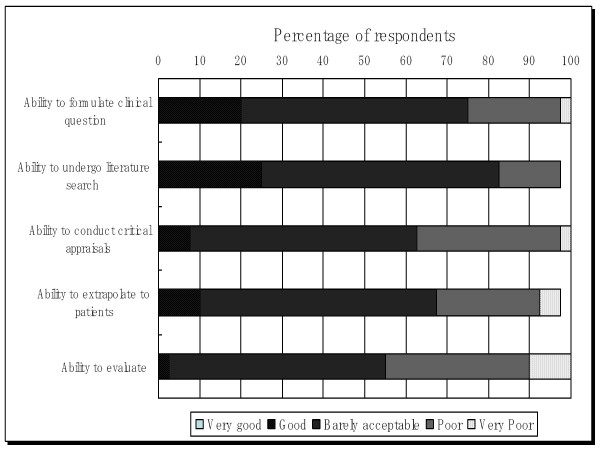
**Respondents' self-rated understanding of their EBM skills**.

Respondents were asked to rate themselves over their confidence in EBM skills. Each item was rated as: very poor ability (score = 1), poor (score = 2), barely acceptable (score = 2), good (score = 4) and very good ability (score = 5). Distinguished skills examined in this survey were formulated from clinical questions and literature searches. This assessment is however subjective since there is no validation study to measure respondents' real competency. Figure 4 presents respondents' self-rated understanding of their EBM skills.

There was a significant correlation between familiarity and use of electronic EBM resources and self-rated understanding of EBM skills (P value for Spearman correlation test = 0.006); however, no other factors showed any significant correlation. Moreover, self-rated understanding of residents with internet access at work did not differ compared to those without (P value for Mann Whitney's comparative test = 0.489).

### Possible Barriers of EBM Application in Daily Practice

**Figure 5:**
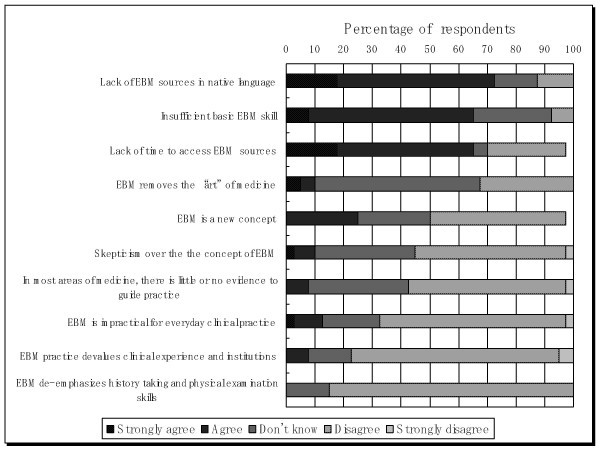
**Respondents' opinion about barrier to implement EBM**.

The three most common barriers to EBM application indicated in this study were as follows: a lack of time to access EBM sources, a lack of sources in the native language, and insufficient skills. No other statements examined in this study were suggested as being barriers to EBM implementation. Figure 5 presents respondents' opinion about barrier to implement EBM.

## Discussion

This survey demonstrated that physician residents at Saga University Hospital positively welcomed EBM promotion. Comparing to others study done in Australia [4], Canada [5], and Saudi Arabia [9], Japanese physicians also showed positive respond and welcomed EBM. The present situation revealed from this study somewhat differs to the comments written by Yokota et.al. (2005); interest in EBM increased significantly during the 1990's but in the mid 2000's EBM concept approval in Japan went through a stagnation period. According to Yokota, the declining acceptance of the EBM concept was caused by the different points of view of EBM pioneers, largely epidemiologists and clinicians, and those directly responsible for clinical EBM practice [12].

Until now, Japanese doctors tended to learn, implement and practice method used by more experienced senior doctors [12]. However, the facts found in this study suggest that they are also beginning to welcome the concept of EBM positively. Thus, it is implied that on one side there is a welcome acceptance of EBM while at the same time maintaining a practice previously established among physicians in Japan.

The most understood EBM resources were shown to be Pubmed, Evidence based medicine (the BMJ publishing group), and Clinical Evidence (the BMJ publishing group); knowledge of other sources was indicated but their utilization during decision making remained rare. It was also found that the electronic resources "Up to Date" and "I Chuu Shi" (Japanese medical search engine) were used by 7.5% and 5% of respondents during clinical decision making.

Factors consider as barrier to implement EBM, there are three predominant factors revealed as barriers to implement EBM in this survey. Those three factor mostly same like others factor found in others study about EBM implementation [13-15]. Barriers related to time constraint are not specific for EBM implementation only, almost all residents' physicians and or all physicians in general experience "time constraint related problem". One thing that interesting found in this study is about the language of EBM source. Language barrier to be one of the three main constraints are found in this study.

### Factors affecting the understanding and use of EBM

This study also revealed that there was no significant mean difference in familiarity of electronic EBM sources between male and female respondents (Mann Whitney U test P = 0.226), residents from surgery and non-surgery groups (Mann Whitney U test P = 0.891), and those with and without Internet facilities at work (Independent sample T test P = 0.228). In addition, there was no difference between groups of residents who work less than 40 hours per week, 41-80 hours per week and more than 81 hours per week, respectively (Kruskal Wallis' test P = 0.283). We also found no correlation between years of residency and familiarity of electronic EBM sources (Spearman's correlation test P = 0.196).

This study also revealed that there was no significant mean difference in understanding of methodological terminology between male and female respondents (Independent sample T test P = 0.679), residents from surgery and non-surgery groups (Mann Whitney U test P = 0.516), and those with and without Internet facilities at work (Independent sample T test P = 0.11). In addition, there was no difference between groups of residents who work less than 40 hours per week, 41-80 hours per week and more than 81 hours per week, respectively (Kruskal Wallis' test P = 0.797). Understanding of methodological terminology also had no correlation with years of residency (Spearman's correlation test P = 0.467).

The only factor apparently influencing the mastery of EBM skills according to the results of this study was familiarity with and use of electronic evidence based sources. The other factors allegedly related to mastery of EBM skills; namely, internet facilities at work, length of residency, and knowledge of the methodological terminology, were not correlated. Of course it should be noted that this assessment is subjective since there is no validation study to confirm knowledge about EBM or to evaluate mastery of methodological terminology.

One of the criticisms often attributed against EBM is concern that doctor experience is considered of no value compared with evidence based facts; however, the facts obtained here seem inconsistent with this criticism. Obstacles in EBM implementation; namely, the fact that the concept of EBM is relatively new, the loss of respect for anamnesis skills and experience of the clinician, impractical application of EBM, a lack of evidence in most areas of the clinic, turned out to be not very influential according to this study.

### Limitations

Despite its findings, this study had its limitations. Namely the design of the study, cross sectional only incorporate one single moment, all of the statement are participant self-report and self-judgement. Other limitation, the sample size was too small for the findings to be generalized.

### Further study

Assessment of the improvement of health care professionals' knowledge and understanding of medical literature, critical appraisal skills, and evidence seeking behaviors is now needed. Determination of an effective way to understand and apply EBM in daily practices is also important.

## Conclusion

In general, attitudes towards EBM are positive. Respondents moderately understood and knew basic EBM skill; however, barriers in its application were shown to exist. Base on these finding we would like to suggest to Saga University teaching hospital to conduct more workshops on EBM, and facilitate residents' physician to improve their comprehensions and skills to EBM.

## List of abbreviations used

EBM: Evidence based Medicine.

## Competing interests

The authors declare that they have no competing interests.

## Authors' contributions

RM: Involved in conception and developing questionnaires, analyzing data, conception, and drafting manuscript. SE: Involved in developing questionnaires, made substantial contribution in distributing the questionnaire and gave advice to content of manuscript. TN: Carried out translation of the questionnaire from English into Japanese version. SK: Involved in developing questionnaire, revising content of result, given final approval of the version to be published, and in charge of whole process as general supervisor of research group. All authors read and approved the final manuscript.

## Supplementary Material

Additional file 1 **Appendix**. Questionnaire for the study titled "Japanese Resident Physicians' Attitudes, Knowledge, and Perceived Barriers on the Practice of Evidence Based Medicine: a Survey". Click here for file
