# Supplementary material for: Japanese Resident Physicians' Attitudes, knowledge, and Perceived Barriers on the Practice of Evidence Based Medicine: a Survey
Source: BMC Res Notes. 2011 Sep 28;4:374. doi: 10.1186/1756-0500-4-374 (PMC3203856; doi:10.1186/1756-0500-4-374)
Supplement: Additional file 1 — Appendix. Questionnaire for the study titled "Japanese Resident Physicians' Attitudes, Knowledge, and Perceived Barriers on the Practice of Evidence Based Medicine: a Survey". [file 1756-0500-4-374-S1.RTF]

Additional Files

Additional File 1: Appendix

Question 1. This section of the questionnaire inquires about residents physicians' preferences of information sources to make clinical decision

There are varieties of information sources to guide clinical decision making. Among the entire sources listed below, please give an X mark which describes your interaction with the entire possible source.
Statement	Always
	Often
	Sometimes
	Seldom
	Never
	
Consult senior doctor directly						
Consult colleagues directly						
Present the case at a problem solving conference to obtain opinion from other doctor/teacher experienced in similar problem 						
Consult clinical practice guidelines 						
Consult resident's manual 						
Attend continual medical education conferences and present your case 						
Read research article						
Read  medical textbook						
Read Today's therapy in particular specialty						
Find out the answer using electronic search engine (Google, yahoo, AltaVista, etc)						


Question 2. This section of the questionnaire inquires about opinion and attitudes towards EBM [9, 10]
We would like to ask you some questions about your current opinion towards Evidence based Medicine, please mark an X on the boxes corresponding to your responses or opinion
Statement	Strongly Agree
	Agree

	Don't Know
	Disagree

	Strongly
Disagree	
EBM practice improves patient care						
EBM improves patient outcomes						
EBM helps clinical decision making						
EBM practice can reduce healthcare costs						
EBM brings about quick knowledge update						
EBM focused on patients value						
EBM should be taught in medical school						
EBM is equal to research activity						
EBM application is difficult in daily practice						


Question 3. This section of the questionnaire inquires about familiarity and use of electronic EBM sources [10]
There are some publication relevant to EBM, please indicates those which you have used or aware of.
EBM Resource	Unaware

	Aware but not use
	Read

	Used to help in clinical Decision making
	
Evidence based medicine (from the BMJ Publishing Group)					
Cochrane database of Systematic Review (part of Cochrane library)					
The American College of Physician Journal Club					
Pubmed/medline journal					
Clinical evidence (from the BMJ Publishing group)					


Question 4. This section of the questionnaire inquires about knowledge of methodological terminology [9, 10]
There are some terms used in EBM paper, please indicate your interaction to them by giving an X mark.
Terminology
used in EBM paper	It would not be helpful for me to understand
	Don't understand but would like to

	Some understanding

	Understand and could explain to others
	
Relative risk					
Absolute risk					
Systematic review					
Clinical effectiveness					
Meta analysis					
Number need to treat					
Odds ratio					
Sensitivity and specificity					
Confidence interval					
Publication bias					
Sample bias					


Question 5. This section of the questionnaire inquires about self-rated confidence in EBM skill [10]
Among all the basic EBM skill please describe your confidence of understanding for each skill:

Statement	Very good
	Good
	Barely acceptable
	Poor
	Very poor
	
Formulate clinical question 						
Literature search 						
Critical appraisal						
Extrapolate to patient						
Evaluation 						


Question 6. This section of the questionnaire inquires about opinion about barrier to implement EBM [10],
There are some possible barrier of EBM application, among all the statement listed below please give an X correspond to your interaction with each statement.

Statement	Strongly Agree
	Agree

	Don't Know

	Disagree

	Strongly
Disagree
	
EBM is a new concept						
EBM practice devalues clinical experience and institutions						
EBM is impractical for everyday clinical practice						
EBM removes the “art” of medicine						
EBM de-emphasizes history taking and physical examination skills						
In most areas of medicine, there is little or no evidence to guide practice						
Lack of time to access EBM sources						
Lack of EBM source in native language						
Insufficiency of basic EBM skill						
Scepticism over the concept of EBM						
